# Ethionamide versus ethambutol-containing first-line regimens for TB meningitis

**DOI:** 10.1128/aac.00190-26

**Published:** 2026-06-15

**Authors:** Xueyi Chen, Carlos E. Ruiz-Gonzalez, Yuderleys Masias-Leon, Medha Singh, Madelynn Shambles, Charles A. Peloquin, Sanjay K. Jain

**Affiliations:** 1 Center for Infection and Inflammation Imaging Research, Johns Hopkins University School of Medicine, Baltimore, Maryland, USA; 2 Center for Tuberculosis Research, Johns Hopkins University School of Medicine, Baltimore, Maryland, USA; 3 Department of Pediatrics, Johns Hopkins University School of Medicine, Baltimore, Maryland, USA; 4 Infectious Disease Pharmacokinetics Laboratory, Pharmacotherapy and Translational Research, University of Florida College of Pharmacy, Gainesville, Florida, USA

**Keywords:** tuberculosis, tuberculous meningitis, chemotherapy, ethambutol, ethionamide

## Abstract

Ethionamide has emerged as an alternative to ethambutol for the treatment of tuberculous meningitis (TB meningitis). Here, we show that ethionamide- and ethambutol-containing first-line TB regimens achieved similar bactericidal activities in brain tissues and reduction in neuroinflammation in a mouse model of TB meningitis. However, ethionamide, but not ethambutol, achieves brain concentrations above the minimum inhibitory concentration for *Mycobacterium tuberculosis*.

Tuberculosis (TB) remains a critical global health concern, and tuberculous meningitis (TB meningitis) represents the most severe form of TB disease. Standard first-line treatment for drug-susceptible pulmonary TB consists of isoniazid, rifampin, pyrazinamide, and ethambutol. While ethambutol performs well in pulmonary lesions (1), its cerebrospinal fluid (CSF) penetration is limited, potentially restricting efficacy in TB meningitis (2). Ethionamide, an inhibitor of mycolic acid synthesis, has emerged as an alternative to ethambutol for TB meningitis because it achieves higher CSF levels (3) and may be associated with improved outcomes (4). We therefore compared the central nervous system exposures, bactericidal activity, and effects on neuroinflammation of ethionamide versus ethambutol-containing first-line TB regimens in a mouse model of TB meningitis.

Female C3HeB/FeJ mice were intracranially inoculated with ~6.4 log_10_ colony-forming units (CFU) of *Mycobacterium tuberculosis* H37Rv (5, 6) and treated following infection with HRZ (isoniazid, rifampin, and pyrazinamide), HRZE (E, ethambutol added), or HRZEt (Et, ethionamide added) (Fig. 1A). Drugs were administered by daily oral gavage (isoniazid 10 mg/kg/day, pyrazinamide 150 mg/kg/day, rifampin 10 mg/kg/day, ethambutol 100 mg/kg/day, and ethionamide 50 mg/kg/day) (7, 8) (Table S1 shows the corresponding human-equivalent doses), 5 days per week with adjunctive dexamethasone (6). Quantification of bacterial burden in the whole brain and lungs was performed at 2 and 6 weeks after treatment. Antibiotic concentration in plasma, brain, lungs, and CSF was determined 2 weeks after treatment (steady state) at near-peak exposure by LC–MS/MS at the Infectious Diseases Pharmacokinetics Laboratory using validated methods, with lower limits of quantification of 0.05 μg/mL for ethambutol and 0.1 μg/mL for ethionamide. Neuroinflammation was assessed by immunofluorescent staining for Iba1 in fixed brain sections and by plasma measurements of glial fibrillary acidic protein (GFAP) via ELISA.

All regimens reduced bacterial burden compared with untreated controls. After 2 weeks of treatment, mean brain bacterial counts (log_10_ CFU/g) were 5.41 for HRZ, 5.35 for HRZE, and 5.09 for HRZEt (HRZE versus HRZEt, *P* = 0.236). At 6 weeks, values declined further to 4.23, 4.57, and 4.06, respectively, with modestly higher activity noted in the ethionamide-containing regimen (HRZE versus HRZEt, *P* = 0.011) (Fig. 1B; Fig. S1A). In the lungs, mean CFU counts were 1.35 for HRZ, 0.51 for HRZE, and 0.38 for HRZEt after 6 weeks of treatment (Fig. 1C; Fig. S1B). Antibiotic levels were measured at steady state after 2 weeks of treatment, at Tmax (15 min for ethionamide [9] and 2 h for ethambutol after oral dosing). The median tissue-to-plasma ratio for ethambutol was 2.24 in the lungs, which is consistent with prior literature (1), and 0.11 and 0.03 in the brain and CSF, respectively. The median tissue-to-plasma ratio for ethionamide was 0.19 in the lungs, and 0.39 and 0.04 in the brain and CSF, respectively (Fig. 2A). Median ethionamide concentrations in the brain (2.16 μg/g) exceeded those of ethambutol (0.38 μg/g) (*P* = 0.166), whereas CSF concentrations were low and comparable between both drugs (0.19 μg/mL and 0.09 μg/mL, respectively) (*P* = 0.392) (Fig. 2B). The low CSF levels of ethionamide observed in our mouse studies, compared to what is noted in humans, are most likely explained by differences in sampling time. Drug levels were measured at the reported plasma Tmax in mice, which is approximately 15 min after oral dosing for ethionamide. However, in the reported human studies, CSF levels were obtained 2–3 h after oral dosing (10). In fact, ethionamide CSF levels during the first hour are below 1 μg/mL in humans (11). When normalized to compound-specific minimum inhibitory concentration (MIC) for *M. tuberculosis* (1 μg/mL for ethionamide and 2 μg/mL for ethambutol), ethionamide achieved higher brain concentration-to-MIC ratios (*P* = 0.024), although CSF ratios were low for both agents (*P* = 0.071). While there was a trend toward lower GFAP levels (Fig. S2) and Iba1-positive staining (marker of microglial activation) (Fig. S3), this was not statistically significant. Finally, although no published studies demonstrate direct synergy between ethionamide and ethambutol, they do target distinct cell wall synthesis pathways, mycolic acid inhibition for the former and arabinogalactan biosynthesis for the latter, raising the possibility of complementary or synergistic effects.

This study has limitations. Antibiotic concentrations were measured at a single timepoint after 2 weeks of treatment, providing steady-state data but not capturing dynamic pharmacokinetic changes over the course of treatment. Model limitations include an artificial route of infection. However, this animal model recapitulates key pathophysiological findings observed in human TB meningitis.

In summary, both ethionamide- and ethambutol-containing regimens achieved similar bactericidal activities in brain tissues, but ethionamide reached substantially higher brain concentrations, aligning with its clinical preference for TB meningitis. Observational studies have reported improved survival when ethionamide replaces ethambutol in pediatric TB meningitis (4). Although ethionamide has adverse effects such as gastrointestinal intolerance and hepatotoxicity, ongoing optimization strategies, including co-administration with EthA activators like alpibectir to reduce dose, may enhance its tolerability (12).

## Supplementary Material

Supp

The following material is available online.

**Data S1 (AAC00190-26-S0001.xlsx).** Source data.

**Supplemental material (AAC00190-26-S0002.docx).** Supplemental methods; Fig. S1 to S3; Table S1.

## Figures and Tables

**FIG 1:**
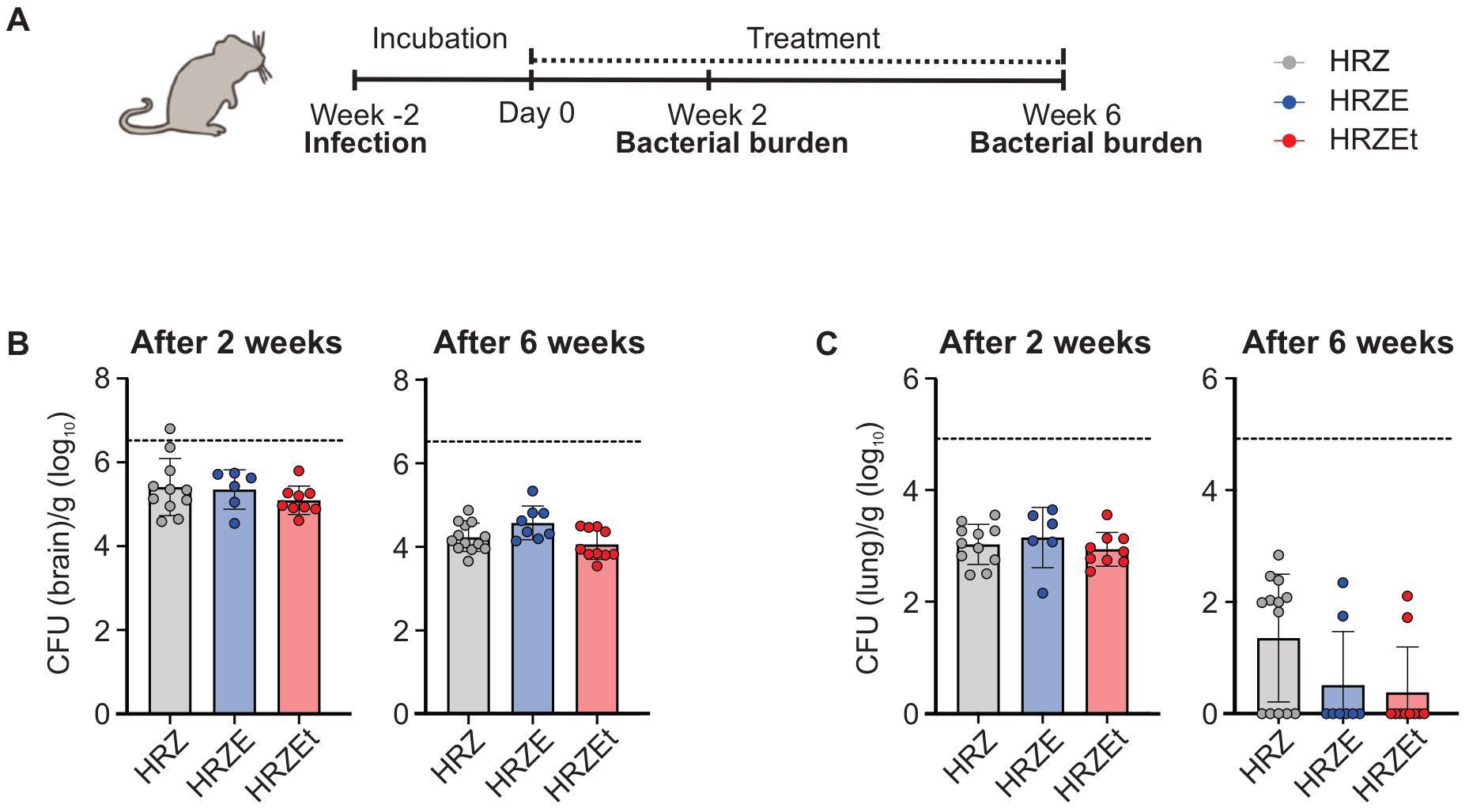
Comparative efficacy of ethambutol- and ethionamide-containing regimens. (A) Mice were randomized to receive the standard HRZ (H, isoniazid; R, rifampin; Z, pyrazinamide), HRZE (E, ethambutol added), or HRZEt (Et, ethionamide added) regimens. (B) Brain bacterial burden (log_10_ CFU/g) after 2 and 6 weeks of treatment. (C) Lung bacterial burden (log_10_ CFU/g) after 2 and 6 weeks of treatment. Data are presented as mean ± standard deviation. The black dashed line represents baseline bacterial burden at treatment start. Each dot represents a single animal, *n* = 6–13 animals per group per timepoint.

**FIG 2:**
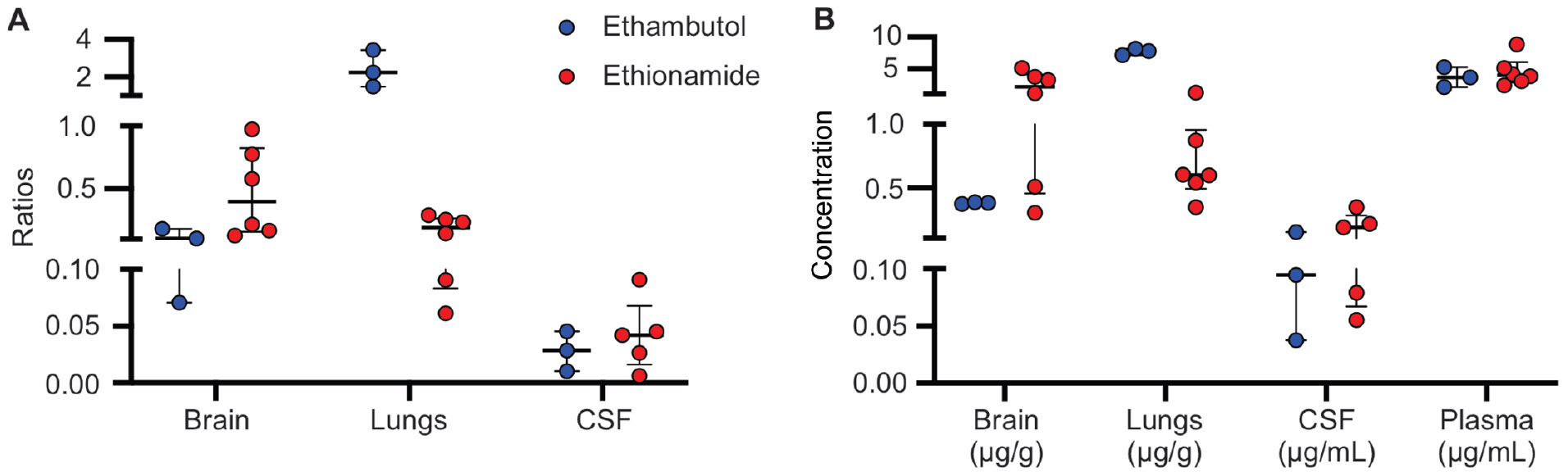
Ethambutol and ethionamide concentrations. (A) Tissue-to-plasma ratios for ethambutol (blue) and ethionamide (red) in the brain, lungs, and CSF. (B) Absolute concentrations in brain and lung tissue (μg/g), CSF (μg/mL), and plasma (μg/mL) after 2 weeks of treatment. Data are presented as median ± interquartile range. Each dot represents a single animal, *n* = 3–6 animals per group.

## Data Availability

All data from this study are present in the paper and/or the Supplementary Materials.
