# Supplementary material for: Ethionamide versus ethambutol-containing first-line regimens for TB meningitis
Source: Antimicrob Agents Chemother. Author manuscript; Available in PMC 2026 Jul 7. (PMC13336335; doi:10.1128/aac.00190-26)
Supplement: Supp [file NIHMS2189112-supplement-Supp.docx]

**Supplementary Material for:**

**Ethionamide versus Ethambutol-Containing First-Line Regimens for TB Meningitis**

Xueyi Chen^1,2,3^, Carlos E. Ruiz-Gonzalez^1,2,3#^, Yuderleys Masias-Leon^1,2,3#^, Medha Singh^1,2,3#^, Madelynn Shambles^1,2,3^, Charles A. Peloquin^4^, Sanjay K. Jain^1,2,3#^*

^1^Center for Infection and Inflammation Imaging Research, Johns Hopkins University School of Medicine, Baltimore, MD, USA.

^2^Center for Tuberculosis Research, Johns Hopkins University School of Medicine, Baltimore, MD, USA.

^3^Department of Pediatrics, Johns Hopkins University School of Medicine, Baltimore, MD, USA.

^4^Infectious Disease Pharmacokinetics Laboratory, Pharmacotherapy and Translational Research, University of Florida College of Pharmacy, Gainesville, FL, USA.

^#^Current affiliation: Department of Pediatrics, Cincinnati Children’s Hospital Medical Center, Cincinnati, OH, USA

*Corresponding author:

Sanjay K. Jain, MD,

Department of Pediatrics, Cincinnati Children’s Hospital Medical Center

240 Albert Sabin Way, MLC7017, Cincinnati, OH 45229, USA

Email: sanjay.jain@cchmc.org

**METHODS**

**Animal infection**

Female C3HeB/FeJ mice, 6-8 weeks old (Jackson Laboratory) were inoculated intracranially with titrated frozen stocks of *M. tuberculosis* H37Rv. Infections were performed through a burr hole (Micro-Drill Kit, Braintree Scientific Inc.), and bacteria were delivered using a Hamilton syringe (Hamilton, 88000) connected to a stereotaxic frame (David KOPF Instruments, model 900) (1, 2). All mice were maintained in a biosafety level-3 (BSL-3) facility with controlled lighting and temperature, without cross-ventilation.

**Assessment of neuroinflammation and injury markers**

Two weeks after treatment completion, animals were euthanized and brains were collected, fixed, and incubated overnight at 4 °C with a primary antibody against ionized calcium-binding adaptor molecule 1 (Iba1) (Thermo Fisher MA5-36257, 1:500). After washing, sections were incubated for 2 hours at room temperature with a goat anti–mouse Alexa Fluor 488 secondary antibody (Thermo Fisher A11034, 1:100), washed, mounted with DAPI-containing antifade medium (Thermo Fisher ProLong Gold Antifade Mountant with DAPI), and imaged at 40× magnification using a Leica DM6 B microscope. For quantitative analysis, ten images per animal were processed in FIJI (ImageJ) to determine the percentage area positive for Iba1 staining. In parallel, plasma samples were collected and stored at −80 °C. The brain injury marker glial fibrillary acidic protein (GFAP) was measured using a commercial ELISA kit (Thermo Fisher EEL098), and values were normalized to total protein content determined by BCA assay.

**Statistical analysis**

Data were analyzed using Prism 10.2.2 (GraphPad). CFU counts are presented on a base-_10_ logarithmic scale as mean ± standard deviation, with between-group comparisons performed using two-tailed Student t tests. All other continuous variables are summarized as median ± interquartile range, and comparisons were performed with two-tailed Mann–Whitney U tests. P values ≤ 0.05 were considered statistically significant.

**FIGURES**

**
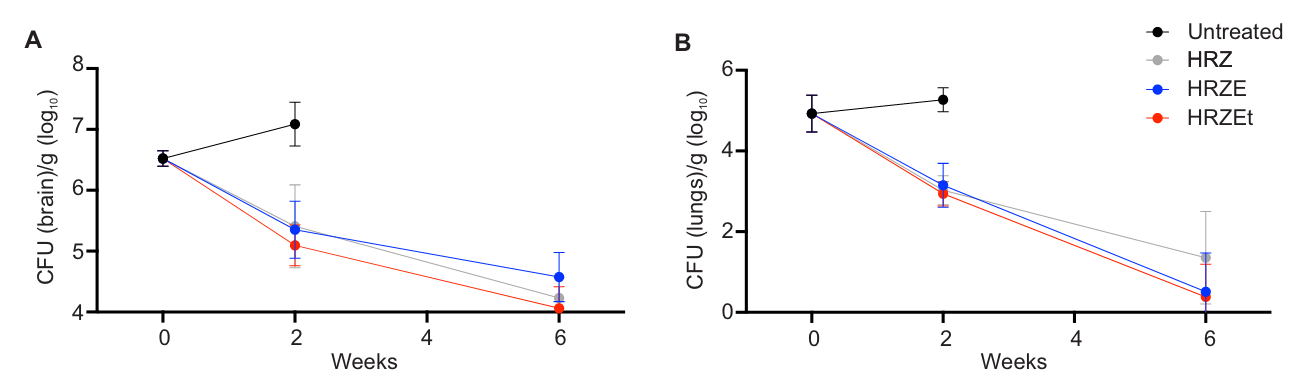
Figure S1. Bactericidal activity.** Brain (**A**) and lung **(B)** bacterial burden (log_10_ CFU/g) in animals treated with the standard HRZ (H, isoniazid; R, rifampin; Z, pyrazinamide), HRZE (E, ethambutol added), or HRZEt (Et, ethionamide added) regimens. Data are presented as mean ± standard deviation.


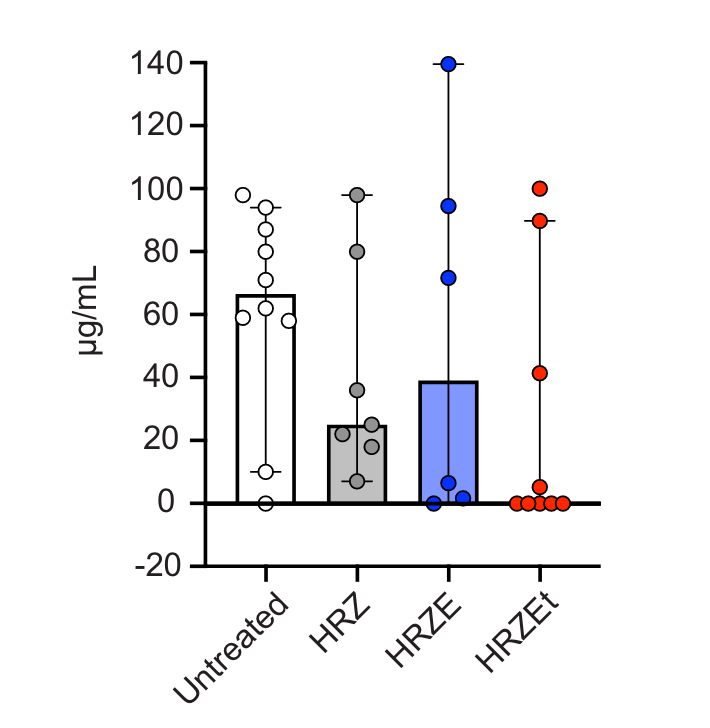


**Figure S2. Serum glial fibrillary acidic protein (GFAP) levels.** GFAP (µg/mL) measured after two weeks of treatment in untreated mice and mice treated with the standard HRZ (H, isoniazid; R, rifampin; Z, pyrazinamide), HRZE (E, ethambutol added), or HRZEt (Et, ethionamide added) regimens. Data are presented as median ± interquartile range. Each dot represents a single animal, n = 6-10 animals per group.


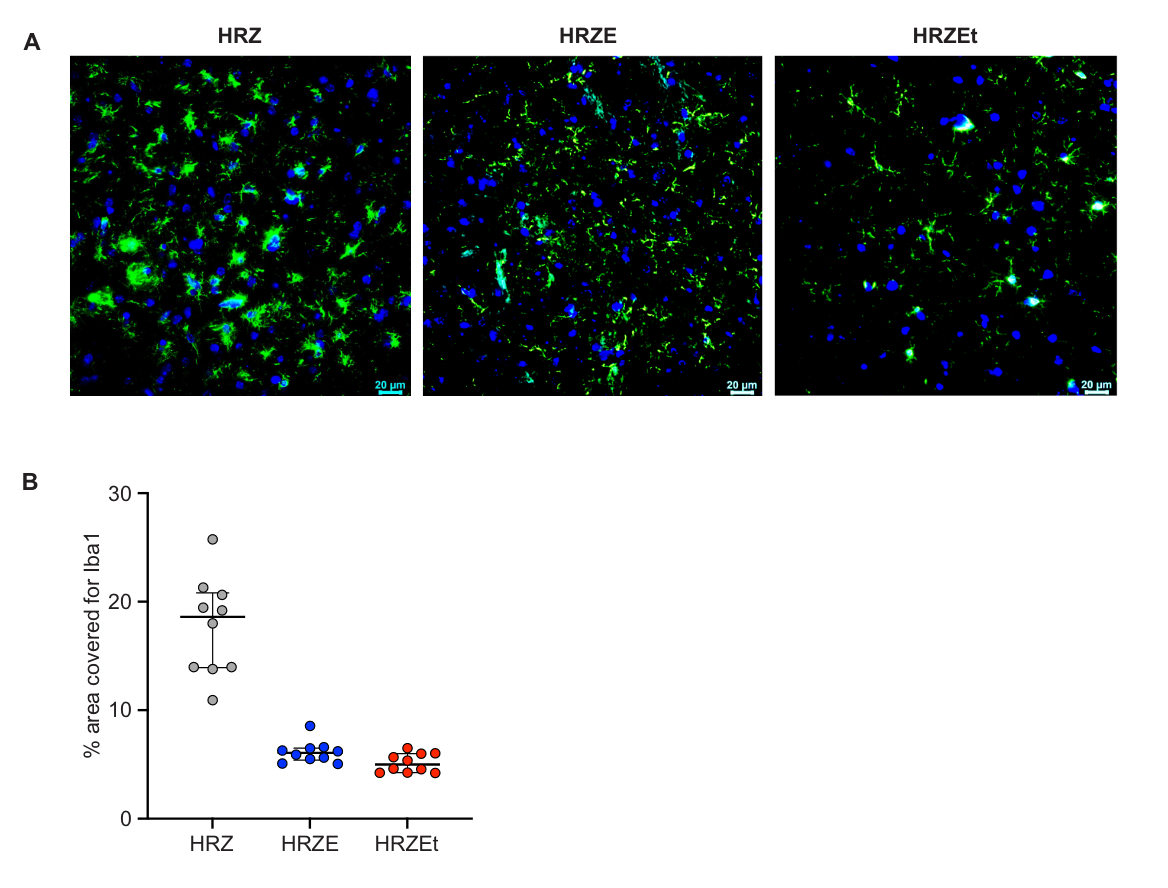


**Figure S3. Microglial activation in brain tissues.** (**A**) Representative brain tissue sections from mice treated for two weeks with the standard HRZ (H, isoniazid; R, rifampin; Z, pyrazinamide), HRZE (E, ethambutol added), or HRZEt (Et, ethionamide added) regimens. The sections are immunostained for Iba1 (green) to visualize microglia, with nuclei counterstained with DAPI (blue). Scale bars, 20 µm. (**B**) Quantification of the Iba1 signal expressed as percent area covered by Iba1. Data are presented as median ± interquartile range. Each dot represents an individual brain section.

**Table S1.** Mouse and the corresponding human equipotent dosing.

| **Drug** | **Mouse dose** | **Human dose** |
| --- | --- | --- |
| Isoniazid | 10 mg/kg | 10 mg/kg |
| Pyrazinamide | 150 mg/kg | 25 mg/kg |
| Rifampin | 10 mg/kg | 10 mg/kg |
| Ethambutol | 100 mg/kg | 15 mg/kg |
| Ethionamide | 50 mg/kg | 15-20 mg/kg (3) |
| Dexamethasone | 2 mg/kg/day | 0.4 mg/kg |

**REFERENCES**

1. Chen X, Arun B, Nino-Meza OJ, Sarhan MO, Singh M, Jeon B, et al. Dynamic PET reveals compartmentalized brain and lung tissue antibiotic exposures of tuberculosis drugs. *Nat Commun.* 2024;15(1):6657.

2. Ruiz-Bedoya CA, Mota F, Tucker EW, Mahmud FJ, Reyes-Mantilla MI, Erice C, et al. High-dose rifampin improves bactericidal activity without increased intracerebral inflammation in animal models of tuberculous meningitis. *J Clin Invest.* 2022;132(6).

3. Thee S, Garcia-Prats AJ, Donald PR, Hesseling AC, and Schaaf HS. A review of the use of ethionamide and prothionamide in childhood tuberculosis. *Tuberculosis (Edinb).* 2016;97:126–36.
